# Effects of Inorganic Salts on Phase Separation in Aqueous Solutions of Poly(ethylene glycol)

**DOI:** 10.3390/ijms26104545

**Published:** 2025-05-09

**Authors:** Pedro P. Madeira, Vladimir N. Uversky, Boris Y. Zaslavsky

**Affiliations:** 1 BiotecFoz, 4250-242 Porto, Portugal; papmadeira@gmail.com; 2 Department of Molecular Medicine and Byrd Alzheimer’s Research Institute, Morsani College of Medicine, University of South Florida, Tampa, FL 33612, USA; 3 Cleveland Diagnostics, 3615 Superior Ave., Cleveland, OH 44114, USA

**Keywords:** aqueous polymer two-phase systems, aqueous polymer–salt systems, phase separation

## Abstract

The effects of a series of sodium salts (Na_3_PO_4_, Na_2_CO_3_, Na_2_SO_4_, Na_2_SO_3_, Na_2_MO_4_, Na_2_CrO_4_, and Na_2_WO_4_) on the phase separation of poly(ethylene glycol) (PEG) solutions in water at PEG concentrations of 0.5 to 30 wt.% were studied. The salts’ effects on phase separation are found to correlate with the change in the entropy related to the structural changes in water during anion hydration. The same salts’ effects on phase separation in aqueous solutions of branched PEG and polyvinylpyrrolidone at a polymer concentration of 10 wt.% were also examined. The results obtained support the assumption that phase separation in aqueous polymer–salt systems is an entropy-driven process.

## 1. Introduction

Partitioning in aqueous polymer two-phase systems is well recognized as a highly efficient and cost-effective method for separating and purifying biological materials [1,2,3]. It can also be used as an extremely sensitive and accurate analytical technique for the analysis of changes in protein structure and protein–protein and protein–ligand interactions [1,2,3] and diagnostics [4].

Aqueous two-phase systems arise in aqueous mixtures of different water-soluble polymers or a single polymer and a specific salt. For example, when polyethylene glycol (PEG) or polyvinylpyrrolidone (PVP) and an inorganic salt, such as potassium phosphate or ammonium sulfate, are mixed in water above certain concentrations, the mixture separates into two immiscible aqueous phases with a clear interfacial boundary. One phase is rich in one polymer, and the other phase is rich in the other polymer or salt. The high water concentration in each of the phases provides a suitable medium for maintaining the tertiary structure of proteins and other biological products. Biological macromolecules, including proteins, enzymes, nucleic acids, etc., are unevenly distributed in such systems, with different components being preferentially retained in one of the phases, providing a basis for separation.

**Figure 1:**
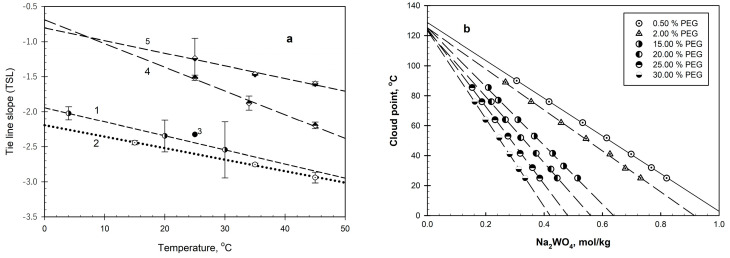

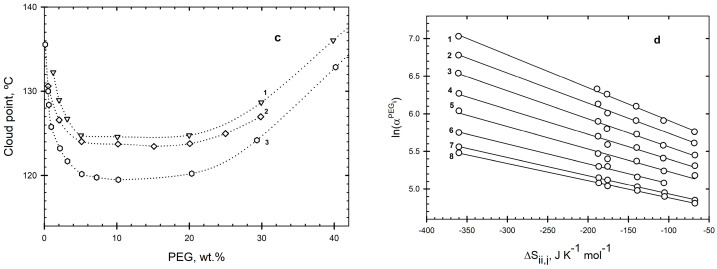
(**a**) Tie-line slopes for PEG-8000%HPO_4_/water systems as functions of temperature. Data calculated from those reported in [6,9,18,19]. (**b**) Cloud point temperature as a function of salt concentration in PEG-Na_2_WO_4_-water systems with various PEG concentrations: 1—0.5 wt.%; 2—2.0 wt.%; 3—15.0 wt.%; 4—20 wt.%; 5—25 wt.%; 6—30 wt.%. (**c**) Cloud point temperature as a function of concentration of PEG in salt-free aqueous solutions: 1—PEG-8000 (data from [40]); 2—PEG-10,000 (obtained by extrapolation, as described in the text); 3—PEG-15,000 (data from [40]). (**d**) The concentration effects of salts, represented by ln(α^PEGij^), on cloud point temperatures in aqueous solutions of PEG as functions of the salt type, quantified by the value of entropy related to the structural changes in water during the hydration of the i-th anion ΔS_ii,j_ at different PEG concentrations (in wt.%): 1—30.0; 2—25.0; 3—20.0; 4—15.0; 5—10.0; 6—5.0; 7—2.0; 8—0.5. Values of ΔS_II,I_ for anions of the sodium salts examined are [41] (in J K^−1^ mol^−1^) as follows: Na_3_PO_4_: 359.4; Na_2_CO_3_: 186.6; Na_2_SO_3_: 174.9; Na_2_MoO_4_: 138.1; Na_2_SO_4_: 126.4; Na_2_SeO_4_: 105.4; Na_2_CrO_4_: 104.2; and Na_2_WO_4_: 65.7.

Multiple studies of aqueous polymer–salt systems were reported in the literature [5,6,7,8,9,10,11,12,13,14,15,16,17]. Ananthapadmanabhan and Goddard [5] showed that phase separation in PEG–salt–water and PVP–salt–water systems is similar to the well-known phenomenon of clouding, which occurs in polymer solutions on heating. The molecular weight distribution characteristics for PEGs used by different authors are likely to vary even when different lots of the same product, e.g., PEG-8000 or PEG-4000, from the same manufacturer are used. Hence, it is hard to compare the results reported by different authors. As an example, Figure 1a shows the tie-line slopes of the phase diagrams for PEG-6000-K_2_HPO_4_–water systems at different temperatures calculated by different authors [6,18,19] as functions of temperature. The tie-line slopes of the phase diagrams calculated from the data reported in the literature [6,7] and presented in the same figure indicate similar trends for different polymers and show that the effect of salt anions is very strong.

Phase separation in aqueous PEG and PVP solutions containing different inorganic salts was examined by multiple authors, but the mechanism of phase separation still remains debatable. It was reported in the literature that the salt effect on the suppression of the cloud point temperature of PEG and PVP in aqueous solutions follows the Hofmeister series, and there were multiple attempts in the literature [20,21,22,23,24,25,26,27,28,29,30,31,32,33] to consider phase separation in aqueous PEG–salt mixtures in terms of salting-out phenomena. The finding [34,35] that PEG forms a two-phase system with NaClO_4_ clearly contradicts this assumption. From the viewpoint of understanding the mechanism of phase separation in polymer–salt ATPS, these attempts, however, may hardly be viewed as successful since the mechanism of the salting-out process remains unknown. According to Pereira and Coutinho [36], the phase separation results from the competition for hydration between the polymer and the salt. Bonnassieux et al. [17] examined the PEG/K_2_HPO_4_ system by multiple techniques to study the solvation properties of both liquid phases and changes in the enthalpy of mixing as a function of ATPS composition. They reported a small enthalpy gain as the driving force for phase separation and hypothesized the entropy of mixing to be small as well.

It has been shown, however, that the salt effect on the phase separation of aqueous PEG solutions correlates linearly with the salt molal surface tension increment [37], which was suggested by Melander and Horvath [38] as a measure of the salt effects on the water structure (i.e., the dynamic and heterogeneous hydrogen-bonded network in liquid water, which includes varying subpopulations with distinct bonding configurations and entropic characteristics). Thiyagarajan et al. [39] reported that the effects of a series of inorganic salts on the phase separation of PEG in H_2_O and D_2_O correlate linearly with the change in water entropy upon the addition of electrolytes. To explore if phase separation in polymer–salt–water systems is entropy driven, as suggested previously [39], we carried out measurements of the phase separation of sodium salt-containing aqueous solutions of PEG-10,000 and PVP-10,000 as a function of anion type.

## 2. Results and Discussion

### 2.1. PEG–Salt–Water Systems

The salt effects on the cloud point temperatures in aqueous PEG solutions at various polymer concentrations are illustrated by the typical data for the PEG-Na2W04-water systems presented in Figure 1b. All the data obtained can be described by:

T_cl.p.ij_ = T^o^_ci.p.j_ − α^PEG^_j_[Salt]_i_, (1)

where T^o^_ci.p.j_ is the cloud point temperature in the salt-free aqueous polymer solution at the polymer concentration of j wt.%; [Salt]_i_ is the concentration of the i-th salt in the polymer solution; T_cl.p.ij_ is the cloud point temperature of the polymer solution at the polymer concentration of j wt.% and at the i-th salt concentration of [Salt]_i_, (in mol/kg); and α^PEG^_ij_ is a constant (slope) characterizing the i-th salt concentration effect upon the cloud point temperature in the aqueous solution of PEG at the PEG concentration of j wt.%.

It is important to notice that the polymer concentration affects both the cloud point temperature in the salt-free aqueous polymer solution, T^o^_cl.p.j_, and the salt concentration effect characterized by the constant α^PEG^_ij_. The cloud point temperatures for the salt-free aqueous polymer solutions, T^o^_cl.p.j_, were estimated by extrapolating the obtained linear curves and averaging over all of the PEG–salt–water systems examined. The data obtained are presented together with those reported by Bae et al. [40] in Figure 1c. There is a good agreement between the data shown.

**Figure 2:**
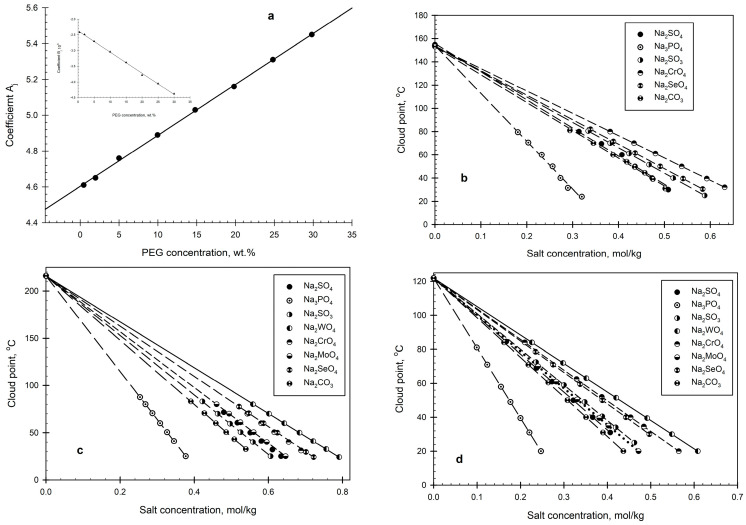
(**a**) Coefficients A and B of Equation (2) as functions of PEG concentration (for an explanation, see the text). (**b**) Concentration effects of inorganic salts, as indicated, on the cloud point of a 10 wt.% aqueous solution of branched poly(ethylene glycol). (**c**) Concentration effects of inorganic salts on the cloud point of a 10 wt.% aqueous solution of polyvinylpyrrolidone. Salts: Na_2_WoO_4_; Na_2_SeO_4_; Na_2_CrO_4_; Na_2_MoO_4_; Na_2_SO_4_; Na_2_SO_3_; Na_2_CO_3_; Na_3_PO_4_. (**d**) Concentration effects of inorganic salts on the cloud point of a 10 wt.% aqueous solution of poly(ethylene glycol). Salts: Na_2_WoO_4_; Na_2_CrO_4_; Na_2_SeO_4_; Na_2_MoO_4_; Na_2_SO_3_; Na_2_CO_3_; Na_2_SO_4_; Na_3_PO_4._ oO_4_; 2—Na_2_CrO_4_; 3—Na_2_SeO_4_; 4—Na_2_MoO_4_; 5—Na_2_SO_3_; 6—Na_2_CO_3_; 7—Na_2_SO_4_; 8—Na_3_PO_4._

The sodium salt concentration effects on the cloud point temperatures in aqueous PEG solutions, as characterized by the slope α^PEG^_ij_, clearly depend on both the PEG concentration and the anion type, as shown by the results presented in Figure 1d. The anion is quantified here by the change in the entropy ΔS_ij_, related to the structural changes in water during the hydration of the anion [41]. The relationships shown in Figure 1d may be described as:

Lnα^PEG^_ij_ = A_j_ + B_j_ΔS_u,i_ (2)

where ΔS_u,i_ is the change in entropy related to the structural changes in water during the hydration of the i-th anion, and A_j_ and B_j_ are constants depending on the PEG concentration j (in wt.%) in the aqueous solution. The polymer concentration affects both constants, as shown in Figure 2a, according to the following relationships:

Aj = 4.605_±0.028_ + 0.0276_±0.0004_C^PEG^ (3)

and

Bj = −2.356±_0.018_ − 0.0703±_0.0011_C^PEG^ (4)

with the correlation coefficients r^2^ = 0.9990 and 0.9986, respectively.

The data shown in Figure 2b indicate unambiguously that the effect of the salt type, as represented by the entropy ΔS_ui_ value, increases with an increasing polymer concentration in the aqueous solution. This result corresponds to the hypothesis [37] that phase separation in PEG–salt–water systems results from the immiscibility of two different water structures arising in a system due to different PEG–water and salt–water interactions. The higher the concentration of PEG, the more significant the difference between the effects of salts with different ΔS_ui_ values on the cloud point temperature (i.e., phase separation). The data obtained here support the conclusion [39] that salt-induced phase separation in aqueous PEG solutions is an entropy-driven process.

To explore if this conclusion is limited to aqueous solutions of PEG only, the salt effects on cloud point temperatures in aqueous solutions of branched PEG and PVP at the polymer concentration of 10 wt.% were examined.

### 2.2. PVP–Salt–Water and Branched-PEG–Salt–Water Systems

**Figure 3:**
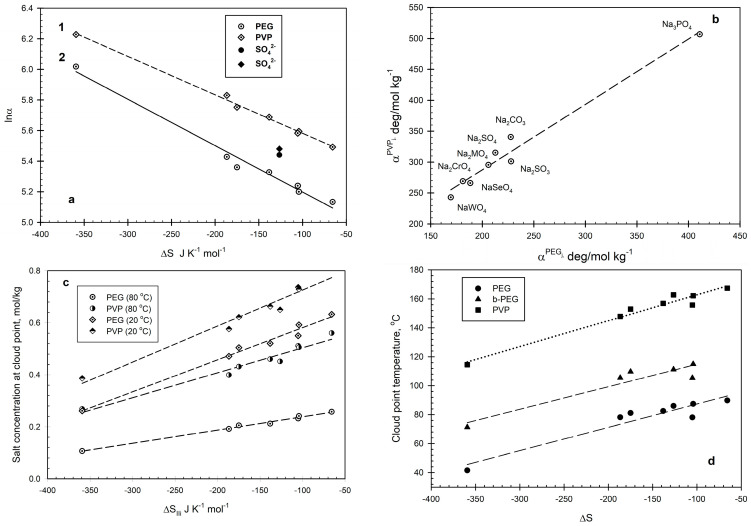
(**a**) Values of α_i_ for PEG (o), b-PEG (Δ), and PVP (♦) solutions as functions of the change in entropy of water at the formation of the hydrated anion, ΔS_ij_. (**b**) Relationship between the α_i_ values for PEG–salt–water and PVP–salt–water systems. (**c**) Salt concentrations required for phase separation in PEG–salt–water and PVP–salt–water systems at 20 and 80 °C as functions of the change in entropy of water at the formation of the hydrated anion, ΔS_ij_. (**d**) Cloud point temperatures in PEG–salt–water, b-PEG–salt–water, and PVP–salt–water systems at the fixed polymer and salt concentrations of 10 wt.% and 0.2 mol/kg, respectively, as functions of the change in entropy of water at the formation of the hydrated anion, ΔS_ij_.

Salt effects on the cloud point temperatures in aqueous b-PEG solutions are shown in Figure 2c, and those for aqueous PVP solutions are shown in Figure 2d. The data on the salt effects in aqueous PEG solutions at the polymer concentration of 10 wt.% are given in Figure 3a for comparison. The T^o^_cl.p_ values obtained are 153.5 ± 2.5 °C for the 10 wt.% b-PEG-10,000 solution, and 216.0 ± 2.0 °C for the 10 wt.% PVP-10,000 solution. The T^o^_cl.p._ value for the aqueous PVP-10,000 solution agrees well with the data reported by Guner and Ataman [42]. The difference between the T^o^_cl.p._ values of about 31 °C for the 10 wt.% solutions of PEG and b-PEG is likely to be due to the more compact structure of branched PEG in water as compared to that of linear PEG.

It can be seen that the salt effects on the cloud point in all the polymer solutions follow approximately the same Hofmeister series. It should be noted that the effects of the same salts on the cloud points in the aqueous PEG and branched PEG solutions are very close. This implies that the conformation of the PEG chain in an aqueous solution is hardly a dominant factor in salt-induced phase separation, as suggested by Karlstrom [43].

The values of Lnα^β^_1_ for the polymer solutions under study are plotted in Figure 3b versus the change in entropy ΔS_u,i_ related to the structural changes in water during the hydration of the i-th anion [41]. The ΔS_u,i_ values for the anions were taken from Krestov [41]. The PEG-Na_2_CO_3_-water and b-PEG-Na_2_SO_4_/water systems, denoted in Figure 3b by filled symbols, do not fit the relationships (see further). The linear relationships observed in Figure 3b indicate that the salt-induced phase separation in aqueous solutions of the polymers under study is indeed an entropy-driven process and is not limited to PEG solutions only.

Comparison of the α^β^_I_ values for the aqueous PEG-salt_i_ and PVP-salt_i_ systems plotted in Figure 3c shows the linear relationship described by:

α^PVP^_i_ = 75.32 + 1.06α^PEG^_i_ (5)

N = 8; r^2^ = 0.9733

where α^PVP^_i_ is the α_i_ value for the PVP (10 wt.%)–salt–water system; α^PEG^_i_ represents the α_i_ value for the PEG (10 wt.%)–salt–water system; r^2^ is the correlation coefficient; and N is the number of polymer–salt–water systems examined. It should be noted that the PEG-Na_2_SO_4_-water system specifically denoted in Figure 3b as not fitting the linear lnα^PEG^_i_–ΔS_u,i_ relationship does not deviate from the relationship described by Equation (5). This probably indicates either that the Na_2_SO_4_ effects on phase separation in both PEG and PVP solutions are salt-specific or, more likely, that the ΔS_u,i_ value for SO_4_ = anion given in [40] is erroneous.

To compare the results in a more straightforward manner, the data are presented in Figure 3c as the salt concentrations required for phase separation in aqueous PEG and PVP solutions at two different temperatures, 20 and 80 °C. The data shown in Figure 3c indicate that the difference in the salt concentrations needed for phase separation in aqueous solutions of both PEG and PVP is more pronounced at a lower temperature and that the anion effect is more significant in the PVP solution than in the PEG solution (at the same polymer concentration of 10 wt.%). Both observations correspond well to the major role of the water structure in the phase separation process.

The current view of the liquid water structure is a uniform, continuous, irregular network of H-bonds with fluctuating lengths of oxygen–oxygen distances, bond angles, and bond energies. It has been shown [44] that the water structure can be described by a set of four different subpopulations. Polymers, such as PEG and PVP, are capable of reorganizing the water structure and changing at least some of the aforementioned features of the water H-bond network [37], resulting in changes in the solvent properties of aqueous media [45]. The influence of PVP on the water structure appears to be stronger than that of PEG, as indicated, for example, by changes in water mobility [46], dielectric properties of aqueous media [47], and solvent properties. Depending on the polymer structure, the size and shape of its macromolecules, and its aggregating ability, a given polymer may affect different features of the water structure to a different degree.

Using the analogy of phase separation in aqueous two-polymer systems [37], it can be suggested that phase separation in a polymer solution results from the polymer-induced redistribution of water subpopulations. The latter term is used here to cover the fraction of the solvent (water) being affected by the polymer to a degree much less, if at all, than the other fraction involved in the polymer hydration shell and serving as the solvent in the polymer-rich phase after phase separation occurred. Different temperature sensitivity of the water subpopulations coexisting in the polymer solution may be the reason for the temperature-induced phase separation. The higher cloud point temperature in the salt-free PVP solution as compared to that in the solution of PEG is likely to be due to the stronger influence of PVP on the coexisting water subpopulations. The salts’ effects on these subpopulations are less significant [48] than those of the polymers. The stronger the polymer influence on the water structure is, the greater the amount of a salt is required to overcome this influence and to impose the salt-specific water structure, immiscible with that imposed by the polymer and leading to phase separation. Since an increasing temperature disrupts the water structure, the salt effects are more pronounced and differentiated at lower temperatures.

The salt effects on phase separation in the aqueous solution of branched PEG (b-PEG) appear to agree with the above model. The cloud point temperatures in the aqueous solutions of the polymers examined at the (randomly selected) salt concentration of 0.2 mol/kg are presented in Figure 3d as functions of the anion type represented by the ΔS_u,i_ value. Phase separation in aqueous polymer solutions at the fixed polymer and salt concentrations occurs at temperatures increasing in the series PEG < b-PEG < PVP. It should be mentioned, additionally, that the relationships established here are similar to those reported in [6], describing the relationship between the cloud points for a series of PEG-3400–salt solutions (at fixed PEG and salt concentrations) in water and D_2_O. This similarity confirms the conclusion that the water structure plays the major role in phase separation in polymer–salt–water systems.

It is important to note that while our study supports entropy-driven separation, the direct observation of salt- or polymer-induced changes in water structure was not conducted here. Techniques such as Raman spectroscopy or neutron scattering, as previously used by Thiyagarajan et al. [39], could serve as powerful tools to validate our thermodynamic interpretations and are recommended for future work. Furthermore, previous calorimetric investigations (e.g., Bonnassieux et al. [17]) revealed only minor enthalpic contributions, reinforcing entropy as the dominant driver. The balance ΔG = ΔH − TΔS supports this view, as ΔH values remain small while phase separation still occurs.

Although only sodium salts were used in this study to maintain consistency, we acknowledge that the role of cations should not be overlooked. Hydration differences among cations, such as Na^+^, K^+^, and NH_4_^+^, can influence phase behavior, and this represents a vital direction for future research. The deviation of Na_2_SO_4_ from the linear trend is potentially due to inaccuracies in the reported entropy of hydration (ΔS_u,i_) for SO_4_^2−^. The independent determination of ΔS_u,i_ through advanced hydration studies could clarify this inconsistency.

The entropy-driven interpretation also suggests changes in the distribution between bound and free water populations. Here, an entropy gain results from the disruption of structured water around polymers and ions, leading to increased microheterogeneity. Although we did not directly measure this ratio, prior work using ¹⁷O NMR and dielectric spectroscopy [44,45,46] supports such a redistribution.

The role of temperature is dual: it disrupts hydrogen bonding and modulates polymer–salt–water interaction competition. With increasing temperature, the differences between salt- and polymer-structured water diminish, reducing the salting-out effect, thereby illustrating how thermal energy alters the balance between polymer- and salt-imposed water structures.

The strength of salt effects on clouding is likely to be linked to the entropy of anion hydration. Structure-making anions (e.g., SO_4_^2−^) and structure-breaking anions (e.g., ClO_4_^−^) interact differently with water subpopulations, leading to differential disruption and, thus, varying cloud point shifts. To illustrate potential applications, we suggest a hypothetical case study: in a separation process requiring mild salting-out, salt like NaClO_4_ with lower ΔS_u,i_ would be preferable over Na_2_SO_4_. Therefore, ΔS_u,i_ can serve as a predictive tool for selecting salts based on the desired separation strength and can be used to predict salting-out efficiency in a hypothetical bioseparation context (e.g., selective precipitation of a protein using PEG–sulfate vs. PEG–perchlorate systems).

The important issue, for the most part, ignored in the current attempts to design theoretical models of phase separation in aqueous polymer solutions, remaining to be resolved, seems to be the reason for the parabolic-like pattern of the cloud point temperature–concentration dependence in the salt-free aqueous polymer solution. Only after this and other issues of phase separation in aqueous solutions of single polymers are explored would it be possible to design a completely self-consistent model of phase separation in polymer–salt–water systems.

## 3. Materials and Methods

### 3.1. Materials

Poly(ethylene glycol) and poly(vinylpyrrolidone), both with the number-average molecular weight (M.) of 10,000, were purchased from Sigma-Aldrich (St. Louis, MO, USA). The so-called branched PEG (8 arm) (b-PEG) of the same number-average molecular weight (M.) of 10,000 was purchased from Shearwater Polymers. The salts, including Na_3_PO_4_, Na_2_CO_3_, Na_2_SO_4_, Na_2_SO_3_, Na_2_CrO_4_, Na_2_SeO_4_, Na_2_WO_4_, and Na_2_MoO_4_, were purchased from Sigma-Aldrich. All salts were of analytical reagent grade. Deionized water with resistivity greater than 18 MQ’cm was used in all experiments.

### 3.2. Measurements

All measurements were performed in triplicate. The standard deviation was within ±3% for the cloud point temperature and ±5% for the salt concentration. Error bars reflecting these variations were added to the revised figures.

Phase separation measurements were carried out over the temperature range of 20 to 85 °C by turbidimetric titration [49] using sealed glass test tubes immersed in a water bath with the temperature regulated within 0–1 °C. The samples were allowed to equilibrate for at least 20 to 30 min between incremental adjustments in temperature. The solutions used in these measurements were prepared by dissolving anhydrous salts in a polymer (PEG, b-PEG, or PVP) solution in water at a fixed polymer concentration. The polymer solution of a given salt was titrated with the corresponding salt-free polymer (PEG, b-PEG, or PVP) solution until the turbid mixture just turned clear at a given temperature. The final mass of the system was determined, and the salt concentration at the point of phase transition was calculated.

## Data Availability

Data made available upon request.
